# Editorial: Combined Therapeutic Approaches to Neurological Rehabilitation

**DOI:** 10.3389/fresc.2022.918005

**Published:** 2022-06-08

**Authors:** Elizabeth Rochon, Gail A. Eskes, Elizabeth R. Skidmore, Carolee J. Winstein

**Affiliations:** ^1^Department of Speech-Language Pathology and Rehabilitation Sciences Institute, University of Toronto, Toronto, ON, Canada; ^2^Toronto Rehabilitation Institute, The KITE Research Institute, University Health Network, Toronto, ON, Canada; ^3^Departments of Psychiatry, Psychology & Neuroscience, Dalhousie University, Halifax, NS, Canada; ^4^School of Health and Rehabilitation Sciences, Department of Occupational Therapy, University of Pittsburgh, Pittsburgh, PA, United States; ^5^Division of Biokinesiology and Physical Therapy, Herman Ostrow School of Dentistry, University of Southern California, Los Angeles, CA, United States; ^6^Department of Neurology, Keck School of Medicine, University of Southern California, Los Angeles, CA, United States

**Keywords:** rehabilitation, neurological disorders, rehabilitation technology, multidisciplinary interventions, motor learning, cognition, communication, pharmacology

In this Frontiers special Research Topic we feature studies that combine intervention approaches to leverage improved rehabilitation outcomes in one or more domains. Studies examine varied combinations of neurophysiological, behavioral and pharmacological interventions, to address a range of cognitive, motor, and communication outcomes. Studies span a variety of neurological populations, functional domains, rehabilitation disciplines, and designs. By their very nature, these multi-component interventions all acknowledge the complexity of functional recovery in rehabilitation, while attempting to uncover underlying neurological and behavioral mechanisms of recovery, capitalize on the opportunity for neuroplasticity and maximize rehabilitation outcomes. The studies have implications for both theoretical, mechanistic accounts of experience-dependent neuroplasticity and for new approaches to interventions in rehabilitation. Several themes emerge from the 10 papers in this special issue.

First, researchers are investigating the effects of combining brain stimulation with cognitive or motor treatments to improve outcomes. Pastore-Wapp et al. describe a study protocol in which repetitive transcranial magnetic stimulation (rTMS) will be combined with video-game-based skill training compared to a video-game-based skill training alone condition. The hypothesis is that the combined condition will better improve dexterity in participants with Parkinson's disease in both the short and long term, leading to improvement in activities of daily living and quality of life. Hildesheim et al. review the factors affecting the use of rTMS for enhancing motor recovery post stroke. This group, The Canadian Platform for Trials in noninvasive Brain Stimulation (CanStim) network, aims to advance the use of rTMS to enhance post stroke recovery by encouraging standardized research protocols in clinical and pre-clinical studies. As such, their paper reviews existing clinical trials for demographic, clinical, and neurobiological factors that predict treatment response. Their review highlights several potential predictive factors. It also highlights the high variability in rTMS protocols and study designs and points to the need to better understand a number of factors, including the mechanisms by which rTMS might enhance recovery and the need for a better of understanding of the combinatorial approach. In a case study combining another neuromodulation technique with speech-language treatment, Figeys et al. employ transcranial direct current stimulation (tDCS) paired with script training for a stroke survivor with aphasia. Although they found a large effect size for the script training alone, the addition of tDCS did not improve script accuracy. However, there was a significant change in the rate of script acquisition. This study's careful single subject design and analysis lead the authors to suggest factors that should be considered in the application of tDCS to aphasia therapy and in future research.

Additional studies examined the use of interactive technologies to promote rehabilitation outcomes. Volk et al. investigated the benefit of an intensive combined electromyography and visual feedback training program for patients with postparalytic facial synkinesis. They showed that facial grading was improved by reducing synkinesis and that effects were durable over 6 months. They suggest that these findings warrant a comparison to other approaches in a future randomized controlled trial; further they highlight the importance of incorporating patient-reported outcome measures in future research. In a similar vein, self-reports from stroke survivors and informal caregivers regarding the use of Socially Assistive Robots in physical therapy were collected in focus groups by Dembovski et al.. Several themes emerged from these very rich, qualitative data, that included both similarities and differences between stroke survivors and caregivers regarding the motivational capabilities of robots in therapy, whether robots are seen as replacements or adjuvants to the clinician, as well as aspects related to technical/ personalization of robots.

Another theme evident in several papers in this issue is combinatorial rehabilitation interventions based upon purported coherent functional networks, domains or systems, resulting in improved performance or outcomes. Vangilder et al.'s secondary analysis of clinical trial data shows that global cognition scores in participants with Parkinson's disease predicted follow up performance in an upper extremity motor task. The implications of this proof-of principle study point to the relationship of cognitive to motor deficits in recovery, and it raises the question of which cognitive deficits might be most related to motor abilities and how combinatorial interventions might best be structured. With the aim of targeting both mood and cognition, Sathananthan et al.'s VaLiANT trial combines cognitive rehabilitation with psychological therapy. This protocol paper outlines a Phase II trial to evaluate a multi-domain intervention for individuals with acquired brain injury. The study will evaluate feasibility as well as a primary outcome of wellbeing and several secondary outcomes, such as cognition, mood and quality of life. Two other studies, both targeting word retrieval in aphasia, focus on a combination of content and process in their respective interventions. In another single subject design, Martin et al. contrast the performance patterns in patients who respond differentially to the linguistic component (i.e., words) vs. the processing component (i.e., response delay) of their treatment, suggesting that personalized treatment based upon accurate diagnosis most likely will lead to better outcomes and provides support for models of speech production that incorporate a verbal short-term memory component of word processing. Simic et al. investigate the feasibility and preliminary efficacy of combined working memory training and targeted anomia therapy in individuals with aphasia, showing that this combination treatment is feasible overall and appears to show transfer to communication contexts beyond single word naming. These authors also suggest that further research is warranted regarding the cognitive abilities that are at play in aphasia therapy at different stages of recovery.

Lastly, Plummer et al. combine a pharmacological intervention (dalfampridine) with physical therapy in individuals with multiple sclerosis in their proof-of-concept study. Results showed that physical therapy combined with medication tended to improve walking function (i.e., gait speed) more than when physical therapy was provided alone. The authors conclude that physical therapy that is based upon motor relearning principles, such as was provided in their study, combined with dalfampridine, warrants further investigation.

This special topics issue well illustrates not only the potential merits of combinatorial approaches, but also the diversity of designs ranging from single-subject, focused reviews, detailed protocols, and small-scale single-site to planned multi-site randomized clinical trials, each reflecting the diverse stage of knowledge development and exciting future trajectory in this topic area. The range of article types reflects perhaps the rather early stage of this effort to move research from a silo perspective to a more multidisciplinary conceptual and collaborative one. If carried forward with the care and thoughtfulness of the projects described here, the combined approach is likely to not only promote new knowledge pertaining to neural and behavioral recovery-supportive mechanisms of neuroplasticity, but also promote more feasible application to current multidisciplinary team approaches. This multidisciplinary team of Guest Editors hopes that this special topics issue triggers new and creative conceptual thinking and sound research in this much-needed area of rehabilitation.

## Author Contributions

ER wrote the first draft of the editorial. GE, CW, and ES contributed to manuscript revision, read and approved the final manuscript. All authors contributed to the conception of the special Research Topic and took individual responsibility for editing separate articles.

## Conflict of Interest

CW serves as a member of the DSMB for Enspire DBS Therapy, Inc., and as a member of the DSMB for Syntactx; a consultant for MicroTransponder, Inc. and receives royalty payments from Human Kinetics, Inc. (6th edition of Motor Control and Learning), and Demos Medical Publishers (2nd edition of Stroke Recovery and Rehabilitation). GE has a patent application pending for an intervention for improving cognitive function. The remaining authors declare that the research was conducted in the absence of any commercial or financial relationships that could be construed as a potential conflict of interest.

## Publisher's Note

All claims expressed in this article are solely those of the authors and do not necessarily represent those of their affiliated organizations, or those of the publisher, the editors and the reviewers. Any product that may be evaluated in this article, or claim that may be made by its manufacturer, is not guaranteed or endorsed by the publisher.

